# Validation of the Scale of Psychological Evaluation Specific to Intensive Therapy (IPAT) on a Population of Patients in Romania Admitted to Intensive Care Units

**DOI:** 10.2478/rjaic-2020-0014

**Published:** 2020-09-27

**Authors:** Stelian Atila Balan, Gabriela Droc, Elisabeta Nita, Dana Tomescu

**Affiliations:** 1 Intensive Care Unit, Fundeni Clinical Institute, Bucharest Romania

**Keywords:** Surgical Trauma, Metabolic Shock, Intramedullary Nailing, Gliding Hip Screw, Minimally Invasive Technique, Interleukins

## Abstract

**Objective:**

Through this study, we want to see to what extent the stress is present among the patients admitted in the intensive care units of the Fundeni Clinical Institute of Bucharest, outlining intervention strategies both individually and collectively and validating the psychological evaluation tool (IPAT) specific to the anesthesia and intensive care units in our population.

**Method:**

Intensive Psychological Assessment Tool (IPAT) with 10 items was used for stress assessment in the intensive care unit and Hospital Anxiety Depression Scale (HADS) with 14 items and two subscales, one for anxiety (7 items) and one for depression (7 items).

**Conclusion:**

The study demonstrates the validity of IPAT scale for the patients participating in the study; the results of the study provide the specialists in anesthesia and intensive care units directions to identify elements of stress, anxiety and depression – directions that can improve their daily work, communication with patients and possibly a better quality of life for all involved in the care of a patient.

**Results:**

The results of the study provide the specialists in anesthesia and intensive care units the directions to improve their daily work and possibly a better quality of life for all involved in the care of a patient.

## Introduction

A person who arrives at an anesthesia and intensive care unit may develop temporary psychological disorders, may experience a dysfunctional condition or even a sudden mental breakdown, resulting in severe psycho-trauma.^[1]^ The emotional states produced by stress involve three components: the subjective experience of the emotion, the tendency to act in a way to overcome the difficulty, the psycho-physiological changes specific to the emotional states.^[2]^ Often patients in the anesthesia and intensive care units develop a UTI syndrome, which is an organic syndrome manifested by a variety of psychological reactions such as fear, anxiety, depression, hallucinations and delirium. The causative factors of this syndrome are: the patient’s medical history prior to admission to the intensive care unit, prior stress adaptation skills, current and previous medication, current clinical status, environmental factors in the intensive care unit. The treatment of UTI syndrome consists of: correction or elimination of the causative factors, choice, dosage and administration of anxiolytic and antipsychotic agents, reduction or elimination of sources of stress related to the environment, frequent communication with the patient and his family.^[3]^

Intensive care units use an ICUSS (intensive care unit stress scale) instrument developed by Dorothy M. Wade, which was derived from a review of the stress and reaction literature in intensive care and has four subscales that include physical stress, delirium symptoms, control and support. In 2014, a shorter form of this IPAT instrument was developed with fourteen elements that take into account the following: communication, breathing difficulties, pain, sleep, anxiety, panic, depression, disorientation, delusions, hallucinations and amnesia.^[4]^

In a 2011 study by Peris Adriano et al., in Italy, in the intensive care unit, they highlighted the importance of psychological intervention for the quality of patients’ lives. Educational interventions, counseling, stress management, psychological support and coping strategies have been used to ease anxiety, depression, fear, helplessness and to reduce discomfort caused by health conditions and medical procedures. Stress management intervention consists of cognitive and emotional restructuring, and interventions are also designed to help family members (starting from the phase when the patient is still unconscious) by promoting the family-centered decision-making process and supporting the parent to choose how well the patient is interacting during their bed visits.^[5]^

In Romania, there is currently no psychological assessment tool specific to the anesthesia and intensive care unit. Through this study, we want to see to what extent the stress is present among the patients admitted in the intensive care units of Fundeni Clinical Institute of Bucharest, outlining intervention strategies both individually and collectively, and validating the psychological assessment tool (IPAT) specific to the anesthesia and intensive care units in our population.

## Methods

The working hypothesis was that stress is present and has an increased level among patients admitted to the intensive care units of Fundeni Clinical Institute of Bucharest, and the study carried out between February 2019 and June 2019 is a study of exploration and quantitative approach to stress. The Intensive Psychological Assessment Tool (IPAT) with 10 items was developed by Dr. Dorothy M. Wade and Hospital Anxiety Depression Scale (HADS) with 14 items was validated on Romanian population by Dr. Maria Ladea.^[6]^ The IPAT scale contains 10 questions about the period spent in the intensive care unit and how it felt during this period, each item has three variants that reflect the severity, rated from 0 to 2, and a total score of over 6 indicates a patient at risk. This scale was translated by two Romanian language translators. The differences between the two translations were discussed, then a Romanian version was finalized. Another authorized translator translated this version into English and there were no understandable differences from the original English version. The test for validation of the IPAT scale was performed on a group of 50 patients, 24 women and 26 men aged 19 to 82 years (M = 56 years ± 16 SD, 95% CI), who were in the intensive care for over 48 hours.

The validity was performed by testing the concurrent validity between the IPAT Scale on one hand and the HADS (Hospital Anxiety and Depression Scale) scale considered “golden standard” on the other. The HADS scale is a two-dimensional 14-item scale that contains two subscales, one for anxiety (7 items) and one for depression (7 items). The HADS scale has been widespread over the past twenty years, is short and is for identifying anxiety and depressive states and the severity of these conditions. The research was approved by the Ethics Commission of the Fundeni Clinical Institute in Bucharest, and the patients answered the two questionnaires during the hospitalization in two intensive care units within the institute together with clinical psychologists. For the statistical analysis of the results obtained from the two tests, the statistical software Epi Info was used, a statistical software developed by the Centers for Disease Control and Prevention (CDC) in Atlanta, Georgia (USA) and licensed as a public domain. The association between the response categories was analyzed using the chi-squared test, the contingency table between the items in the IPAT scale. With logistic regression, the interaction between the dependent variable (the score for each item in the IPAT scale) and the independent variables (the score for each item in the HADS scale) was analyzed. The logistic model analyzes data using the maximum likelihood estimation method, the maximum fidelity and the results obtained are statistically significant p < 0.05. *Odds ratios* are determined by probabilities and range from 0 to infinity. Odds are defined as the ratio of probability of success and probability of failure. 95% - error margin for C.I.; *C.I*. - confidence interval with some uncertainty in the estimation; *Coefficient* - the regression coefficient that represents the mean of changing the response variable for a single unit of change in the predictor variable while keeping other predictors in the model constant; *S.E*. - the standard error is an estimate of the standard deviation of a coefficient; *Z-statistic* - is the regression coefficient divided by its standard error*; P-value* - a predictor with a low p-value is a significant addition to the model because the variations of the predictor value are correlated with the changes of the response variable; *Constant* - guarantees that the residues do not have a positive or negative overall tendency and serve as a waste bin for any prejudice that is not explained by the terms in the model.

## Results

For the fidelity of the IPAT test, the internal consistency (α Cronbach) was calculated. The internal consistency (α Cronbach) of the original scale is 0.8 (Dorothy M. Wade, 2014), and the internal consistency on the Romanian version of the scale (α Cronbach) is 0.75.

A total score on the IPAT scale of over six points indicating a patient at risk was found in a number of 20 patients, is 40% of those who participated in the study. The items on the IPAT scale with the highest score are: item 2 – “Was it difficult to sleep?”, item 3 – “Did you feel stressed?” and item 4 – “Did you feel sad?” A statistically significant association was established between item 1 of the IPAT scale – “ Was it difficult to communicate?” – and item 2 of the IPAT scale – “Was it difficult to sleep?” having p = 0.003 (p < 0.05, chi-squared test, degrees of freedom df = 4) (Table 1).

**Table 1:** Association of items in the IPAT-contingency table

		Was it difficult to communicate?		
Was it dificult to sleep?	**0**	**1**	**2**	**Total**
**0**	5	1	1	7
Row%	71.43%	14.29%	14.29%	100.00%
Col%	21.74%	6.25%	9.09%	14.00%
**1**	16	7	2	25
Row%	64.00%	28.00%	8.00%	100.00%
Col%	69.57%	43.75%	18.18%	50.00%
**2**	2	8	8	18
Row%	11.11%	44.44%	44.44%	100.00%
Col%	8.70%	50.00%	72.73%	36.00%
**TOTAL**	23	16	11	50
Row%	46.00%	32.00%	22.00%	100.00%
Col%	100.00%	100.00%	100.00%	100.00%

Row% - percentage of Chi-Squared df Probability total item “Was it difficult 15.7287 4 0.0034 to sleep?” for each answer

Col% - percentage of total item “Was it difficult to communicate?” for each answer

The interaction between the dependent variables was analyzed, item 1 of the IPAT scale “*Was it difficult to communicate?*”, item 5 of the IPAT scale “*Did you feel panicked?*”, item 6 of the IPAT scale “*Did you feel hopeless?*” and all independent variables in the anxiety subscale and depression subscale of the HADS scale using the logistic regression model, and the result obtained is statistically significant p = 0.000 (p < 0.05, likelihood test) and indicates a risk factor for anxiety (Table 2) and depression for patients in intensive care units (Table 3).

**Table 2:** Analysis of risk factors through the logistic regression model Item 1 – “*Was it difficult to communicate?*” and all items in the anxiety subscale

Term	Odds Ratio	95%	C.I.	Coefficient	S. E.	Z-Statistic	P-Value
I feel tense or nervous	2.5772	0.4370	15.1999	0.9467	0.9054	1.0456	0.2958
I have a feeling of fear that something very bad is about to happen	2.8021	0.7684	10.2176	1.0304	0.6601	1.5609	0.1185
I worry	0.4547	0.0991	2.0867	-0.7881	0.7774	-1.0137	0.3107
I can be calm and feel relaxed	1.7984	0.3035	10.6558	0.5869	0.9078	0.6465	0.5179
I have a feeling of fear as if I have a knot in my stomach	4.1177	0.8312	20.3974	1.4153	0.8164	1.7336	0.0830
I feel the need to move as if I couldn't stay	1.3271	0.5507	3.1983	0.2830	0.4488	0.6307	0.5283
I suddenly feel panic	1.1116	0.2439	5.0660	0.1058	0.7739	0.1368	0.8912
**CONSTANT**	*	*	*	-3.7651	1.2897	-2.9194	0.0035

Item 5 – “*Did you feel panicked?*” and all items in the anxiety subscale							

**Term**	**Odds Ratio**	**95%**	**C.I**.	**Coefficient**	**S. E**.	**Z-Statistic**	**P-Value**
	
I feel tense or nervous	55.6798	2.0297	1527.4674	4.0196	1.6897	2.3789	0.0174
I have a feeling of fear that something very bad is about to happen	0.3523	0.0519	2.3922	-1.0433	0.9773	-1.0675	0.2857
I worry	0.4630	0.0781	2.7435	-0.7700	0.9078	-0.8482	0.3963
I can be calm and feel relaxed	4.1581	0.3159	54.7226	1.4250	1.3149	1.0837	0.2785
I have a feeling of fear as if I have a knot in my stomach	5.6272	0.6578	48.1410	1.7276	1.0952	1.5775	0.1147
I feel the need to move as if I couldn't stay	1.5898	0.5288	4.7796	0.4636	0.5616	0.8255	0.4091
I suddenly feel panic	3.8353	0.4474	32.8781	1.3443	1.0962	1.2263	0.2201
**CONSTANT**	*	*	*	-7.3094	2.4566	-2.9754	0.0029

Item 6 - “*Did you feel hopeless?*” and all items in the anxiety subscale							
**Term**	**Odds Ratio**	**95%**	**C.I**.	**Coefficient**	**S. E**.	**Z-Statistic**	**P-Value**
**I feel tense or nervous**	2.8722	0.3028	27.2487	1.0551	1.1479	0.9191	0.3580
**I have a feeling of fear that something very bad is about to happen**	0.8783	0.1714	4.4995	-0.1298	0.8336	-0.1557	0.8763
**I worry**	1.4491	0.2555	8.2196	0.3709	0.8855	0.4189	0.6753
**I can be calm and feel relaxed**	10.4606	0.6913	158.2872	2.3476	1.3861	1.6936	0.0903
**I have a feeling of fear as if I have a knot in my stomach**	6.3208	0.9502	42.0456	1.8439	0.9668	1.9072	0.0565
**I feel the need to move as if I couldn't stay**	1.6644	0.6014	4.6063	0.5094	0.5194	0.9808	0.3267
**I suddenly feel panic**	0.8198	0.1207	5.5671	-0.1987	0.9773	-0.2033	0.8389
**CONSTANT**	*	*	*	-6.8959	2.0803	-3.3148	0.0009

**Table 3:** Analysis of risk factors through the logistic regression model Item 1 - “*Was it difficult to communicate?*” and all items in the depression subscale

Term	Odds Ratio	95%	C.I.	Coefficient	S. E.	Z-Statistic	P-Value
**I like what I liked before**	1.1682	0.2508	5.4417	0.1555	0.7850	0.1981	0.8430
**I can laugh and see the good side of things**	2.0070	0.4390	9.1764	0.6967	0.7755	0.8983	0.3690
**I’m well-disposed**	3.6091	0.7280	17.8924	1.2835	0.8168	1.5713	0.1161
**I feel like I’m doing everything slower**	2.5657	0.8517	7.7296	0.9422	0.5627	1.6746	0.0940
**I’m no longer interested in how I look**	0.8683	0.3025	2.4925	-0.1412	0.5380	-0.2625	0.7929
**I’m glad before when I think I’ll do certain**	0.2742	0.0530	1.4177	-1.2939	0.8382	-1.5436	0.1227
**things**							
**I enjoy watching a TV show, listening to a radio show or reading a good book**	2.9564	0.3227	27.0868	1.0840	1.1302	0.9591	0.3375
**CONSTANT**	*	*	*	-2.5316	1.0327	-2.4514	0.0142

Item 5 – “*Did you feel panicked?*” and all items in the depression subscale							

**Term**	**Odds** **Ratio**	**95%**	**C.I**.	**Coefficient**	**S. E**.	**Z-Statistic**	**P-Value**
	
**I like what I liked before**	0.9285	0.1937	4.4514	-0.0741	0.7997	-0.0927	0.9261
**I can laugh and see the good side of things**	1.0668	0.2475	4.5987	0.0646	0.7455	0.0867	0.9309
**I’m well-disposed**	2.1827	0.4559	10.4509	0.7806	0.7991	0.9768	0.3287
**I feel like I’m doing everything slower**	2.9955	1.0113	8.8725	1.0971	0.5540	1.9803	0.0477
**I’m no longer interested in how I look**	0.8477	0.2809	2.5580	-0.1652	0.5635	-0.2932	0.7694
**I’m glad before when I think I’ll do certain things**	1.7463	0.2962	10.2943	0.5575	0.9052	0.6159	0.5380
**I enjoy watching a TV show, listening to a radio show or reading a good book**	0.6817	0.0880	5.2795	-0.3832	1.0444	-0.3669	0.7137
**CONSTANT**	*	*	*	-2.3287	0.9859	-2.3620	0.0182

Item 6 – “*Did you feel hopeless?*” and all items in the depression subscale							

**Term**	**Odds** **Ratio**	**95%**	**C.I**.	**Coefficient**	**S. E**.	**Z-Statistic**	**P-Value**
	
**I like what I liked before**	1.2321	0.2067	7.3451	0.2087	0.9109	0.2291	0.8188
**I can laugh and see the good side of things**	1.1004	0.1883	6.4323	0.0957	0.9009	0.1062	0.9154
**I’m well-disposed**	6.9489	0.9293	51.9636	1.9386	1.0265	1.8885	0.0590
**I feel like I’m doing everything slower**	2.4512	0.7322	8.2062	0.8966	0.6165	1.4543	0.1459
**I’m no longer interested in how I look**	2.7740	0.6107	12.5992	1.0203	0.7721	1.3214	0.1864
**I’m glad before when I think I’ll do certain things**	0.9046	0.0910	8.9910	-0.1002	1.1717	-0.0855	0.9318
**I enjoy watching a TV show, listening to a radio show or reading a good book**	0.5530	0.0383	7.9881	-0.5925	1.3625	-0.4348	0.6637
**CONSTANT**	*	*	*	-4.8241	1.4853	-3.2479	0.0012

## Discussions

In 2004, Carich and Spilman formulated twelve principles of crisis intervention that are valid for any theoretical model^[7]^:

1. Respect: the level of attention paid according to the patient’s experiences and the uniqueness of the patient
2. The report: creating a harmonious relationship based on trust, sharing, understanding and acceptance
3. To be with the patient: identifying the patient’s values ​and judgments, accepting them as valid and justified
4. Compassion: the interaction with the patient’s subjectivity in the affective, cognitive and behavioral plane
5. Multilevel communication
6. Cooperation
7. Flexibility
8. Use: continuous process of using data obtained from the patient through observation and evaluation of symptoms, behaviors, affective and cognitive expressions
9. Safety: addressing the vulnerability of the patient at the physical and psychological level and inducing the necessary changes
10. Generating change
11. The principle of metaphor: the use of metaphor to increase the motivation for change
12. Goal orientation

Well-managed stress can be overcome; during stressful periods, it is not the stressful stimulus that is psychological, but the way the stimulus is perceived as stressful.^[8]^ In 2018, in Greece, a conclusion of a study was the following: given the high scores of depressive and anxiety symptoms of patients’ relatives and their positive attitudes towards the support of specialists, efforts should be made to respond to these needs through psychological intervention offered by specialists and supportive intervention offered by the entire care team.^[9]^

The purpose of a 2016 study was to evaluate the epidemiology and post-intensive care intervention (ICU), the interventions for the symptoms of anxiety after critical illness. Five databases (1970–2015) were searched to identify studies evaluating anxiety symptoms in adult UTI survivors. Data obtained from studies using the most common assessment tool were meta-analyzed. 27 studies (2880 patients) were identified out of the 27,334 cited. The hospital anxiety subclass (HADS-A) was the most commonly used instrument (81% of studies). Data were collected at time points of 2–3, 6 and 12–14 months, with prevalence of anxiety symptoms (HADS-A ≥ 8, 95% confidence interval [CI]) of 32% (27–38% ), 40% (33–46%) and 34% (25–42%), respectively. In a subgroup of studies with repeated assessments in the same patients, there was no significant change in anxiety score or prevalence over time. One third of UTI survivors have symptoms of anxiety that persist in the first year of recovery. Age, sex, severity of illness, diagnosis and length of stay were not associated with anxiety symptoms. Psychic symptoms during admission and memories of delusional experiences in the ICU were potential risk factors. Psycho-emotional rehabilitation and ICU journals had a potential benefit.^[10]^

In the studies from 2013 and 2015, the following conclusions were highlighted, namely: survivors of intensive care units frequently present with symptoms of anxiety, depression and post-traumatic stress disorder during recovery and that more than two thirds of visiting family members in the intensive care unit had symptoms of anxiety or depression, the prevalence of symptoms of anxiety and depression being high at the end of the stay in the intensive care units, regardless of whether the patient is well enough to be discharged or near death.^[11,12]^ Moreover, in 2014, a study showed that the families of patients who survived with a short stay in the intensive care units may have a similar prevalence of anxiety and depression at the discharge as compared to the families of patients with longer stay in intensive care units.^[13]^

Thus, multidisciplinary follow-up after intensive therapy may be useful for identifying untreated physical and psychological problems for survivors in intensive care units and liaising with specialists helps to resolve identified problems.^[14]^

In 2019, the care guide for patients admitted to the anesthesia and intensive care units highlights the role of the psychologists both for the care team in terms of prevention of burnout and for patients and their families in terms of reducing anxiety, depression, acute stress and post-traumatic stress.^[15]^

A 2015 study showed that IPAT correlates with HADS and highlighted the need for additional research to manage anxiety and depression^[16]^ and our study highlights the risk factors for patients in the anesthesia and intensive care units, elements of anxiety and depression by using the IPAT scale specific to the anesthesia and intensive care units.

## Conclusions

The study demonstrates the validity of the IPAT scale for the patients participating in the study. The results of the study provide specialists in the anesthesia and intensive care units directions to identify elements of stress, anxiety and depression, directions that can improve their daily work, communication with patients and possibly a better quality of life for all involved in the care of a patient.
